# The Skin Secretion of the Amphibian *Phyllomedusa nordestina*: A Source of Antimicrobial and Antiprotozoal Peptides

**DOI:** 10.3390/molecules18067058

**Published:** 2013-06-17

**Authors:** Guilherme D. Brand, Raimunda C. Santos, Luisa Mayumi Arake, Valdelânia G. Silva, Leiz M. C. Veras, Vladimir Costa, Carlos Henrique N. Costa, Selma S. Kuckelhaus, José Guilherme Alexandre, Maria J. Feio, José Roberto S. A. Leite

**Affiliations:** 1 Laboratório de Espectrometria de Massa, EMBRAPA, Recursos Genéticos e Biotecnologia, Brasília, DF 70770-900, Brazil; E-Mails: gdbrand@gmail.com (G.D.B.); luisa.mayumi@gmail.com (L.M.A.); 2 Núcleo de Pesquisa em Biodiversidade e Biotecnologia, Biotec, *Campus* Parnaíba, Universidade Federal do Piauí, UFPI, Parnaiba, PI 64202-320, Brazil; E-Mails: raimundaphb@hotmail.com (R.C.S.); valdelaniabio@hotmail.com (V.G.S.); leiz.veras@gmail.com (L.M.C.V.); vladimir.costa@gmail.com (V.C.); 3 Bolsista de Desenvolvimento Científico Regional, DCR, FAPEPI/CNPq, *Campus* Parnaíba, Universidade Federal do Piauí, UFPI, Parnaiba, PI 64202-320, Brazil; 4 Programa de Doutorado em Biotecnologia, RENORBIO, Universidade Federal do Piauí, UFPI, Teresina, PI 64001-020, Brazil; 5 Laboratório de Pesquisas em Leishmanioses, Instituto de Doenças Tropicais Natan Portela, IDTNP, Teresina, PI, 64001-450, Brazil; E-Mail: chncosta@gmail.com; 6 Laboratório de Imunologia Celular, Área de Patologia, Faculdade de Medicina, Universidade de Brasília, UnB, Brasília, DF 70910-900, Brazil; E-Mail: selmask@gmail.com; 7 REQUIMTE, Departamento de Química e Bioquímica, Faculdade de Ciências da Universidade do Porto, Rua do Campo Alegre 4169-007 Porto, Portugal; E-Mails: jguilhermealexandre@gmail.com (J.G.A.); mffeio@fc.up.pt (M.J.F.)

**Keywords:** antimicrobial peptides, dermaseptins, phylloseptins, *Phyllomedusa*, *Leishmania*

## Abstract

Antimicrobial peptides (AMPs) from the dermaseptin and phylloseptin families were isolated from the skin secretion of *Phyllomedusa nordestina*, a recently described amphibian species from Northeastern Brazil. One dermaseptin and three phylloseptins were chosen for solid phase peptide synthesis. The antiprotozoal and antimicrobial activities of the synthetic peptides were determined, as well as their cytotoxicity in mouse peritoneal cells. AMPs are being considered as frameworks for the development of novel drugs inspired by their mechanism of action.

## 1. Introduction

Antimicrobial peptides (AMPs) are the primary effectors of the innate immunity of amphibians, hindering the colonization of frogs’ skin by invading pathogens [1]. These peptides are active against a wide range of microorganisms such as bacteria, viruses, yeasts and filamentous fungi, and most of them do not present significant toxicity in mammalian cells [2,3,4]. Moreover, AMPs also kill medically relevant protozoa such as *Trypanosoma cruzi*, *Plasmodium falciparum* and various species from the *Leishmania* genus [5,6]. Their antimicrobial action is mostly exerted by a preferential accumulation on the microorganisms’ membranes, followed by the induction of defects and disruption of the cellular osmotic gradient, ultimately leading to cell lysis [7]. Molecules inspired on the structure and mechanism of action of AMPs are being considered for diverse biotechnological applications and for pharmaceutical development [8,9].

The skin secretions of frogs from the Phyllomedusinae subfamily are treasured as a rich source of AMPs, especially molecules from the dermaseptin and phylloseptin families [10,11]. These peptides are small, with no more than 35 amino acid residues, linear, cationic and fold into amphiphilic helices upon contact with the plasma membrane [12,13,14,15,16,17,18]. Reported herein are the isolation and sequencing of AMPs from the skin secretion of *Phyllomedusa nordestina*, a recently described species of the *Phyllomedusa hypochondrialis* group distributed on the Caatinga region and its areas of influence in Northeastern Brazil [12]. The *in vitro* antimicrobial potential of DRS-H10, the shortest dermaseptin ever described, as well as of three phylloseptins, was investigated against the promastigote and amastigote forms of *Leishmania amazonensis* and *Leishmania infantum* (synonymous of *L. chagasi* [19]), as well as representative species of bacteria. The effect of the peptides on the viability of mouse peritoneal macrophages was additionally assayed as a model of toxicity to mammalian cells.

## 2. Results and Discussion

**Figure 1:**
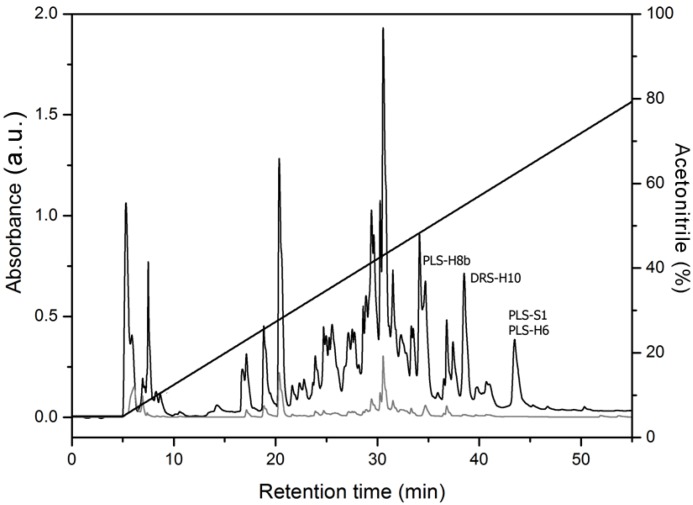
Fractionation of the lyophilized crude extract of the total skin secretion from *P. nordestina*. The elution of the peptides was performed using a linear gradient of H_2_O and acetonitrile supplemented with 0.1% (v/v) trifluoroacetic acid at a 2.5 mL/min flow rate. The absorbance was monitored simultaneously at two wavelengths, 216 nm (black line) and 280 nm (grey line).

The chromatographic analysis of the skin secretion of *P. nordestina* yielded more than forty fractions which were subjected to MALDI-TOF/TOF mass spectrometry for the identification and sequencing of the molecules (Figure 1).

Table 1 lists molecules from the dermaseptin, phylloseptin and hyposin families, their corresponding monoisotopic molecular masses and similarity to peptides found in other phyllomedusid species. The novel peptide nomenclature was adopted [20]. Five dermaseptins were identified, DRS-O1, DRS-H9, DRS-H15, DRS-H3 and DRS-H10, along with the phylloseptins PLS-H5, PLS-H6, PLS-S1, a fragment of PLS-H8 (named PLS-H8b) and the hyposins HPS-H2 and HPS-J1 (Table 1).

**Table 1:** Dermaseptins, phylloseptins and hyposins isolated from the skin secretion of *P. nordestina*.

Primary structure		Exp. [M+H]^+^	Peptide name(s)	Ref.
**Dermaseptins**				
**1**	**GLWSTIKQKGKEAAIAAAKAAGQAALNAASEAL-NH_2_**	**3208.63**	**DRS-H9**	**[6]**
2	GLWSTIKQKGKEAAIAAAKAAGQAALNAASEAL-NH_2_	3208.92	-	-
3	WSTIKQKGKEAAIAAAKAAGQAALNAASEAL-NH_2_	3038.70		
4	TIKQKGKEAAIAAAKAAGQAALNAASEAL-NH_2_	2765.52		
**5**	**GLWSTIKQKGKEAAIAAAKAAGQAALGAL-NH_2_**	**2793.78**	**DRS-01, DRS-H7**	**[14]**
6	GLWSTIKQKGKEAAIAAAKAAGQAALGAL-NH_2_	2793.75	-	-
7	GLWSTIKQKGKEAAIAAAKAAGQAALG-OH	2610.64		
8	TIKQKGKEAAIAAAKAAGQAALGAL-OH	2350.40		
9	WSTIKQKGKEAAIAAAKAAGQAALGAL-NH_2_	2623.49		
10	WSTIKQKGKEAAIAAAK-COOH	1801.18		
**11**	**GLWSKIKDVAAAAGKAALGAVNEAL-NH_2_**	**-**	**DRS-H15**	**[15]**
12	GLWSKIKDVAAAAGKAALNAVNEAL-NH_2_	2480.55	-	-
13	WSKIKDVAAAAGKAALNAVNEAL-NH_2_	2310.40		
**14**	**GLWSTIKNVGKEAAIAAGKAALGAL–NH_2_**	**2409.41**	**DRS-H3, DRS-H12**	**[6]**
15	GLWSTIKNVGKEAAIAAGKAALGAL-NH_2_	2409.55	-	-
16	GLWSTIKNVGKEAAIAAGKAALGAL-OH	2410.55		
17	WSTIKNVGKEAAIAAGKAALGAL-NH_2_	2238.25		
18	TIKNVGKEAAIAAGKAALGAL-NH_2_	1966.23		
**19**	**GLWSTIKNVAAAAGKAALGAL-NH_2_**	**-**	**DRS-H10**	**[15]**
20	GLWSTIKNVAAAAGKAALGAL-NH_2_	1982.26	-	-
**Phylloseptins**				
**21**	**FLSLIPHAINAVSAIAKHF-NH_2_**	**2048.25**	**PLS-H5**	**[15]**
22	FLSLIPHAINAVSAIAKHF-NH_2_	2048.38	-	-
23	LIPHAINAVSAIAKHF-NH_2_	1701.00		
24	IPHAINAVSAIAKHF-NH_2_	1587.88		
25	FLSLIPHAINA-OH	1195.71		
**26**	**FLSLIPTAINAVSALAKHF-NH_2_**	**2012.12**	**PLS-H6**	**[15,16]**
27	FLSLIPTAINAVSALAKHF-NH_2_	2012.36	-	-
28	LSLIPTAINAVSALAKHF-NH_2_	1865.19		
29	SLIPTAINAVSALAKHF-NH_2_	1752.14		
30	LIPTAINAVSALAKHF-NH_2_	1665.17		
31	IPTAINAVSALAKHF-NH_2_	1551.91		
**32**	**FLSLLPSLVSGAVSLVKKL-OH**	**1970.43**	**PLS-H8**	**[16]**
33	FLSLLPSLVSGAVSLVKK-OH	1858.23	-	-
34	SLLPSLVSGAVSLVKKL-NH0_2_	1710.19		
**35**	**LLGMIPVAISAISALSKL-NH_2_**	**-**	**PLS-S1**	**[17]**
36	LLGMIPVAISAISALSKL-NH_2_	1796.18	-	-
**Hyposins**				
**37**	**LRPAFIRPKGK-NH_2_**	**1280.87**	**HPS-H2**	**[18]**
38	LRPAFIRPKGR-NH_2_	1309.95	-	-
39	RPAFIRPKGR-NH_2_	1196.87		
**40**	**FRPALIVRTKGK-NH_2_**	**1383.80**	**HPS-J1**	**[19]**
41	LRPALIVRTKG-OH	1223.90	-	-

**Footnote**: **DRS-H9** (Uniprot Dep. nr. P84880; Seq. method: MS/MS; Species: *P. hypochondrialis*), **DRS-01/DRS-H7** (Uniprot Dep. nr. P83637; Seq. method: Edman, MS/MS; Species: *P. oreades*, *P. hypochondrialis*), **DRS-H15** (Uniprot Dep. nr. P84937; Seq. method: cDNA; Species: *P. azurea*), **DRS-H13/DRS-H12** (Uniprot Dep. nr. P84596.1/Q1EJP5.1; Species: *P. hypochondrialis*, *P. azurea*), **DRS-H10** (Uniprot Dep. nr. Q17UY8.1; Seq. method: cDNA; Species: *P. azurea*), **PLS-H5** (Uniprot Dep. nr. P86710.1/P85882.1/P85447.1; Species: *P. palliate, P. azurea, P. tomopterma*), **PLS-H6** (Uniprot Dep. nr. Q0vz41/P85883/CAJ76135; Seq. method: cDNA, MS/MS; Species: *P. hypochondrialis*, *P. azurea*), **PLS-H8** (Uniprot Dep. nr. Q0vz39; Seq. method: cDNA, MS/MS; Species: *P. hypochondrialis*), **PLS-1** (Uniprot Dep. nr. CAP17494.1; Seq. method: cDNA; Species: *P. sauvagii*), **HPS-H2** (Uniprot Dep. nr. P84955; Seq. method: MS/MS; Species: *P. azurea*), **HPS-J1** (Uniprot Dep. nr. P86613; Seq. method: MS/MS; Species: *P. jandaia*).

**Figure 2:**
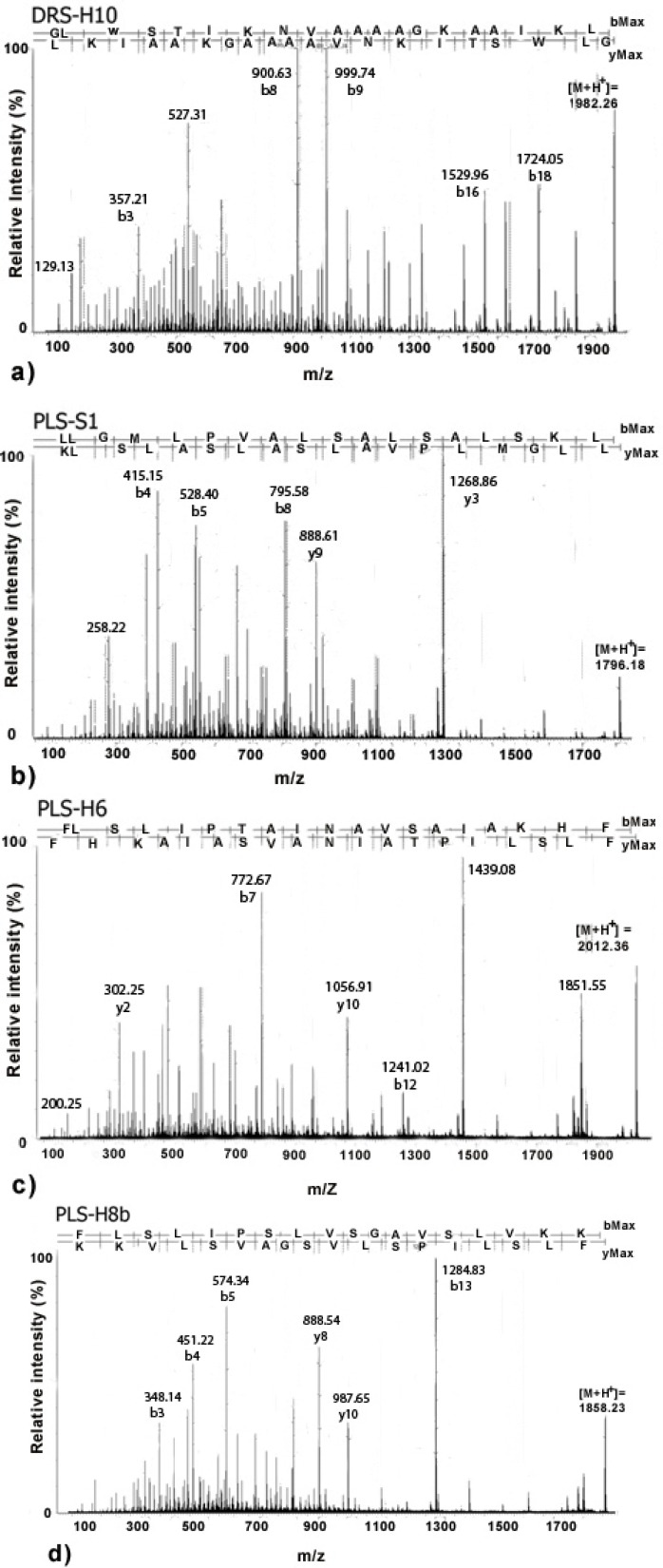
*De novo* sequencing of peptides on the skin secretion of *P. nordestina* (DRS-H10 (**a**), PLS-S1 (**b**), PLS-H6 (**c**), and PLS-H8b (**d**)). The observed fragments allowed complete assignment of the major y and b ion series. The peptide sequence using one-letter code following the y and b series orientation is shown on the top part of the graphs.

The MS/MS spectra of (a) DRS-H10 ([M+H]^+^ = 1982.26 Da), (b) PLS-S1 ([M+H]^+^ = 1796.18 Da), (c) PLS-H6 ([M+H]^+^ = 2012.36 Da) and (d) PLS-H8b ([M+H]^+^ = 1858.23 Da) are depicted in Figure 2. These peptides were chosen for solid phase peptide synthesis and antimicrobial activity evaluation.

The AMPs on the skin secretion of *P. nordestina* are mostly identical to those found in other species of the *P. hypochondrialis* group, such as *P. azurea*, *P. hypochondrialis*, *P. oreades* and *P. rohdei*, which concurs with the hypothesis of recent speciation events [10,12,20]. Partial N- and C-terminal digestions of dermaseptins, phylloseptins and hyposins were abundant in the skin secretion of *P. nordestina*, as listed in Table 1. Although common in other hylid species, such as *Leptodactylus syphax* and *Hypsiboas raniceps*, this is the first time that a range of partially degraded peptides was detected in phyllomedusids [21,22]. We cannot ascertain if such a number of partial degradations is natural to this particular species or a response to a physiological stress for this population. 

Moreover, degradation might arise from inadequate sample handling and storage, although frog secretions were processed similarly in previous studies without the described effect [6,22]. It has been suggested that peptide degradation at specific cleavage sites might increase the molecular diversity of the amphibians’ skin secretions without gene duplication events, conferring protection against a wider range of microorganisms [22]. However, the peptide fragments detected herein seem to indicate the action of unspecific amino- and carboxy-peptidases rather than a controlled mechanism of hydrolysis of specific peptide bonds.

It is known that peptides from the dermaseptin and phylloseptin families are efficient leishmanicidal agents [5,6,23]. To assess the antimicrobial potential of synthetic DRS-H10, PLS-S1, PLS-H6 and PLS-H8b, increasing concentrations of peptides were incubated in vitro with *L. amazonensis* and *L. infantum* promastigote and amastigote cells, followed by the assessment of the protozoan cells’ viability. 

**Figure 3:**
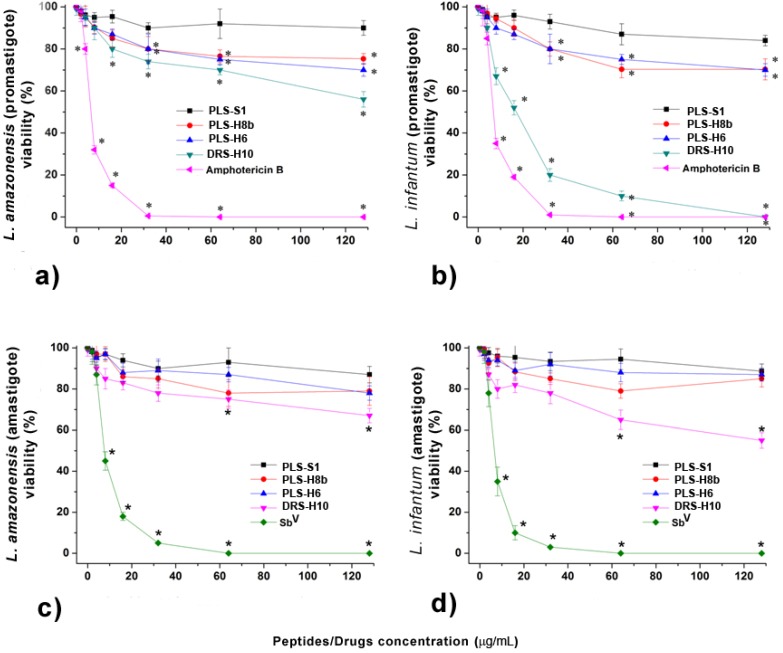
Percentage of living *Leishmania (L.) amazonensis* (**a**) and *L. infantum* (**b**) promastigotes incubated with different concentrations of peptides or amphotericin B for 6 h. Percentage of living *Leishmania (L.) amazonensis* (**c**) and *L. infantum* (**d**) amastigotes incubated with different concentrations of antimicrobial peptides and SbV (Glucantime^®^) for 6 h. Schneider medium was used as control. All data are represented by the mean value and standard deviation. (*) *p* < 0.05 when compared to the control.

Figure 3a,b demonstrate that except for PLS-S1, all other peptides significantly decreased the viability of *L. amazonensis* and *L. infantum* promastigotes at concentrations higher than 32 μg/mL (approximately 16–18 μM, depending on the individual peptide). DRS-H10, in particular, was as potent as amphotericin B, killing half of the population of *L. infantum* promastigotes (IC50) at a concentration of 8.1 μM (16 μg/mL) against the concentration of 9.2 μM (10 μg/mL) required for the reference promastigote-form drug (Figure 3b).

However, the amastigote forms of both assayed *Leishmania* species was more resistant to the action of antimicrobial peptides, while still susceptible to Glucantime^®^ (SbV, meglumine antimoniate). DRS-H10 was the only peptide to decrease the viability of the *L. infantum* amastigotes to approximately 50% and only doing so at a concentration of 64.6 μM (128 μg/mL) (Figure 3c,d). Overall, the *P. nordestina* peptides DRS-H10 and PLS-S1, -H6 and -H8b are considerably less potent agents against *L. amazonensis* and *L. infantum* promastigotes than other peptides described in the literature [5].

Minimal inhibitory concentrations of DRS-H10 against *Escherichia coli*, *Staphylococcus aureus* and the phytopathogenic bacterium *Xanthomonas axonopodis* pv. glycines were determined as 17.8 ± 5.1, 32.3 and 2.0 µM. DRS-O1, the reference peptide, presented MICs of 11.5 ± 5.7, 5.7 and 1.42 µM for the same microorganisms. DRS-H10 was first detected as a transcript in the skin of *P. azurea*, and with 21 amino acid residues, it is the shortest dermaseptin ever described [15]. Considering that it is 84% identical to DRS-H3, it is possible to speculate that its reduced size is responsible for the lower antimicrobial activity. Another possible contributing factor to DRS-H10’s lower efficacy when compared to DRS-O1 is its lower net formal charge, +2 for the first and +4 for the latter, including the amidated C-terminus. Peptide charge is an important factor in the interaction with the negatively charged lipid membrane of the bacterial cell [7].

**Figure 4:**
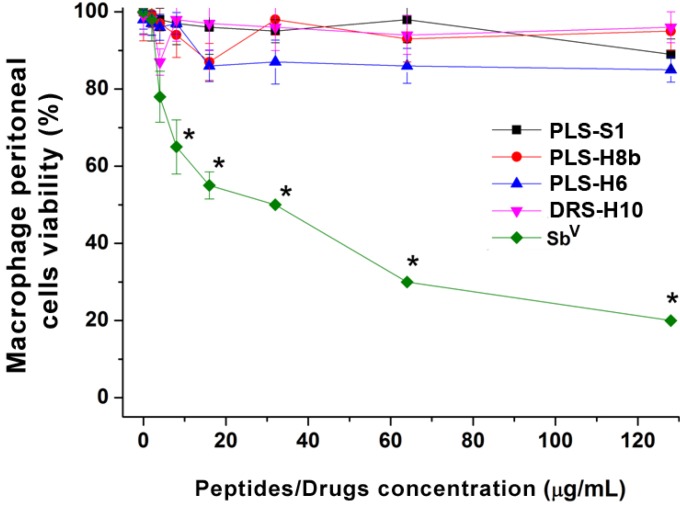
Effect of antimicrobial peptides from *Phyllomedusa nordestina* on macrophage peritoneal cells as measured by the MTT assay. The dispersion bar represents the standard deviation of three independent experiments with reproducible results. (*) *p* < 0.05 when compared to the control.

The synthetic dermaseptins and phylloseptins from *P. nordestina* were incubated with mouse peritoneal macrophages to evaluate possible toxic effects to mammalian cells. Figure 4 shows that none of the tested peptides reduced the macrophages cell viability up to a concentration of approximately 64 µM, contrasting with the pentavalent antimonium compound used as reference (Sb^V^), which presented a LC_50_ at an approximate concentration of 87.4 µM (32 µg/mL).

The lack of toxicity of DRS-H10, PLS-S1, PLS-H6 and PLS-H8b in mouse peritoneal macrophages indicates that although these molecules are not particularly potent leishmanicidal agents, their favorable therapeutic index might make them promising lead compounds for further pharmaceutical development.

Moreover, the reduced size of these molecules makes them promising alternatives in biotechnological applications such as the detection of protozoa in biological fluids by biosensors [9]. To further explore the potential therapeutic use of the dermaseptins and phylloseptins showing promising profiles in this study, additional work is required, including the evaluation of the possibility of chemically modifying these antimicrobial peptides. Only in this way could their specificity be improved, further improving also relevant aspects regarding the safety and efficacy of new therapies for the control of visceral leishmaniasis.

## 3. Experimental

### 3.1. Amphibians

Frog skin secretions were obtained from adult specimens of *Phyllomedusa nordestina*, collected at the Delta do Parnaíba region, city of Ilha Grande, Piauí State, Brazil. Frogs were captured according to the protocols approved by Instituto Chico Mendes de Conservação da Biodiversidade (ICMBio), under process license number 17687-1/2009. Frogs from the same *P. nordestina* population were collected twice in one year interval.

### 3.2. Frog Skin Secretion Fractionation, Peptide Sequencing and Similarity Searches

*Phyllomedusa nordestina* skin secretion was collected as previously described [6]. Skin secretions were immediately frozen after collection. The lyophilized extract was ressuspended in 0.1% TFA (v/v) and purified using a reverse-phase Vydac 218TP510 on a Shimadzu CLASS-LC 10VP instrument (Shimadzu Corp., Kyoto, Japan). Peptides were purified on a linear gradient from 0 to 70% acetonitrile (AcN), with H_2_O:0.1% TFA or AcN:0.1% TFA as mobile phases. When necessary, chromatographic fractions were further purified using analytic columns and optimized gradients. Mass spectrometric measurements were performed on a MALDI TOF/TOF Bruker Ultraflex III (Bruker Daltonics, Billerica, MA, USA) using α-cyano-4-hydroxycinnamic acid as the ionization matrix as described in detail elsewhere [24]. Some amino acid sequencing was performed by the automated EDMAN degradation method on a PPSQ-23 protein peptide sequencer (Shimadzu Co., Kyoto, Japan). Isobaric amino acid residues, such as Leucine/Isoleucine and Lysine/Glutamine were not discerned and their identities are based on sequence similarity to previously identified molecules. Similarity searches were conducted using BLAST (http://blast.ncbi.nlm.nih.gov/Blast.cgi) and FASTA (http://www.ebi.ac.uk/Tools/sss/fasta/) [25]. 

### 3.3. Solid Phase Peptide Synthesis

C-terminally amidated peptides, such as DRS-H10 (GLWSTIKNVAAAAGKAALGAL), PLS-S1 (LLGMIPVAISAISALSKL) and PLS-H6 (FLSLIPTAINAVSALAKHF), were synthesized manually by the Fmoc/*t*-butyl chemistry using a Fmoc-PAL-PEG-polystyrene resin (NovaBiochem^®^, San Diego, CA, USA). PLS-H8b (FLSLLPSLVSGAVSLVKK) was synthesized using an Fmoc-Lys-NovaSyn TGT resin, resulting in a free carboxy terminus after peptide cleavage from the resin. Resin cleavage and peptide purification were conducted as previously described [22].

### 3.4. Leishmania Isolates

The isolate MHOM/BR/pH8 of *Leishmania (L.) amazonensis* was obtained from the Laboratory of Dermatology, Faculty of Medicine, University of Brasilia and the isolate of *L. infantum* was obtained from the Laboratory of Leishmaniasis of the Instituto de Doenças Tropicais Natan Portella (IDTNP), Teresina, Piauí, Brazil. Specimens were kept cryo-preserved in liquid nitrogen and grown by transfer to NNN medium [26] supplemented with LIT (Liver Infusion Tryptose, DIFCO, Detroit, MI, USA) and incubation at 22 °C for 48 h. A small aliquot was added to Schneider insect medium (Sigma-Aldrich, St. Louis, MO, USA) supplemented with 20% (v/v) heat-inactivated fetal calf serum and gentamycin sulphate (40 mg/mL) (Schering Plough, São Paulo, Brazil), and cultured until the log phase was reached.

### 3.5. *In Vitro* Evaluation of the Effect of the Antimicrobial Peptides on the *L. amazonensis* and *L. infantum*

Cell viability was assessed according to the MTT method [26]. To assess the effect of the AMPs DRS-H10, PLS-S1, PLS-H6 and PLS-H8b on *L. amazonensis* or *L. infantum*, 1 × 10^6^ promastigotes or axenic amastigotes were cultured in Schneider insect medium (Sigma-Aldrich) with peptide concentrations ranging from 1 to 128 μg/mL in 96-well microplates, in triplicate, at 22ºC for 2 h. After incubation, 10 μL of 3-(4,5-dimethylthiazol-2-yl)-2,5-diphenyltetrazolium Bromide (MTT) were added to all wells and the plate was re-incubated for 4 h to allow the reduction of MTT by the mitochondria. Subsequently, 50 μL of sodium dodecyl sulfate [10% (w/v) in water] were added to the wells to dissolve the formazan crystals formed and the absorbance of the solution was measured at 570 nm in a SpectraMax® Plus 384 spectrophotometer (Molecular Devices Corporation, Sunnyvale, CA, USA). The leishmanicidal reference drugs amphotericin B and meglumine antimoniate (Glucantime^®^) were purchased from Sigma Chemicals (Saint Quentin Fallavier, Lyon, France) and Rhône-Poulenc-Rorer Laboratories (Montrouge, France, batch number 331-2) respectively, and were used as controls. The reduction of MTT to formazan takes place only when reductase enzymes are active therefore, chemical conversion was used as a measure of viable (living) cells.

### 3.6. Cytotoxic Effects of Antimicrobial Peptides on Peritoneal Cells

Peritoneal cells were obtained by washing the peritoneal cavity of Swiss mice (n=10) with 10 mL of cold PBS, pH 7.2. Recovered macrophages were washed with cold PBS (400 x *g*, 10 min), quantified using a haemocytometer and suspended into cold RPMI 1640 medium (Sigma), pH 7.2, supplemented with 20 mM Hepes (Sigma), 2 mM glutamine (Sigma) and 2.5 µg/mL gentamycin. Viability was assessed with 0.05% (w/v) nigrosin solution in 0.15 M PBS, pH 7.2 [27], and was always higher than 97%. Viable cells (2.5 × 10^4^) were cultured in 96 well microplates for 1 h with different concentrations of peptides (1 to 128 μg/mL) at 37 °C and 5% CO_2_. Cell viability was assessed according to the MTT method [26] as described in the previous section.

### 3.7. Antimicrobial Susceptibility Testing

Antimicrobial susceptibility was assessed for Clinical and Laboratory Standards Institute (CLSI) reference strains *Escherichia (E.) coli* ATCC 25922 and *Staphylococcus (S.) aureus* ATCC 25923. Minimal Inhibitory Concentrations (MICs) were determined in Iso-Sensitest broth (Oxoid, Basingstoke, UK) following a standard micro-dilution technique [28]. A bacterial suspension of 10^8^ CFU/mL was initially prepared in 0.85% (w/v) sterile saline and diluted to 5 × 10^5^ CFU/mL in each microplate well. DRS-H10 was co-incubated with bacteria at concentrations ranging from 1 to 128 μg/mL in two-fold dilutions. Microplates were placed on a wet cotton-bed in closed plastic containers, incubated at 37 °C and read after 18–24 h. Each assay was repeated at least three times with independent starting bacterial inocula. Inhibitory assays for *Xanthomonas axonopodis* *pv. glycines* were conducted by the same methodology using Mueller-Hinton Broth (DIFCO) with an incubation time of 48 h.

### 3.8. Statistical Analysis

The resulting data from the in vivo studies were also analyzed with the non-parametric ANOVA (Kruskal-Wallis) test. Values of *p* < 0.05 were considered to be statistically significant.

## 4. Conclusions

In this work we show that five novel dermaseptins, DRS-O1, DRS-H9, DRS-H15, DRS-H3 and DRS-H10, the phylloseptins PLS-H5, PLS-H6, PLS-S1, a fragment of PLS-H8 (named PLS-H8b) and the hyposins HPS-H2 and HPS-J1 were found in crude extract of the total skin secretion from *P. nordestina*, and identified. With this, this paper reports the first study of peptidome bioactive peptides in relation to this species of frog. All peptides were tested against *L. amazonensis* and *L. infantum* but only the dermaseptin DRS-H10 was better than the reference drug amphotericin B, killing fifty percent of the *Leishmania* population tested at 8.1 µM concentration. This study found that despite the fact several antimicrobial peptides (e.g., dermaseptins and phylloseptins) demonstrate potent antimicrobial activity, they did not show strong antimicrobial action against the bacteria *E. coli*, *S. aureus* and *X. axonopodis*, although they showed low cytotoxicity against mammalian cells in models of peritoneal macrophages.
